# Reduced Graphene Oxide as a Platform for the Immobilization of Amino-Cyclodextrins

**DOI:** 10.3390/mi14040746

**Published:** 2023-03-28

**Authors:** Elias Villalobos, José F. Marco, Claudia Yáñez

**Affiliations:** 1 Centro de Investigación de Procesos Redox, CIPRex, Facultad de Ciencias Químicas y Farmacéuticas, Universidad de Chile, Sergio Livingstone 1007, Independencia, Santiago P.O. Box 233, Chile; 2 Instituto de Química Física “Rocasolano”, CSIC, C/Serrano, 119, 28006 Madrid, Spain; jfmarco@iqfr.csic.es; 3 Departamento de Química Orgánica y Fisicoquímica, Facultad de Ciencias Químicas y Farmacéuticas, Universidad de Chile, Sergio Livingstone 1007, Independencia, Santiago P.O. Box 233, Chile

**Keywords:** graphene, reduced graphene oxide, cyclodextrin, supramolecular construction, graphene platform

## Abstract

In the present work, we reported on a method to combine amino β-cyclodextrins (CD1) with reduced graphene oxide (obtained by the electrochemical reduction of graphene oxide, erGO) to produce a glassy carbon electrode (GCE) modified with both CD1 and erGO (CD1-erGO/GCE). This procedure avoids the use of organic solvents such as hydrazine or long reaction times and high temperatures. The material combining both CD1 and erGO (CD1-erGO/GCE) was characterized by SEM, ATR-FTIR, Raman, XPS, and electrochemical techniques. As proof-of-concept, the determination of the pesticide carbendazim was carried out. The spectroscopic measurements, especially XPS, proved that CD1 was covalently attached to the surface of the erGO/GCE electrode. The attachment of cyclodextrin at the reduced graphene oxide produced an increase in the electrochemical behavior of the electrode. The cyclodextrin-functionalized reduced graphene oxide, CD1-erGO/GCE, showed a larger sensitivity (1.01 μA/μM) and a lower limit of detection for carbendazim (LOD = 0.50 μM) compared with the non-functionalized material, erGO/GCE, (sensitivity = 0.63 μA/μM and LOD = 4.32 μM, respectively). Overall, the results of the present work show that this simple method is suitable to attach cyclodextrins to graphene oxide, maintaining their inclusion abilities.

## 1. Introduction

Graphene is an allotrope of carbon constituted by a two-dimensional monolayer of carbon atoms forming a hexagonal lattice. Graphene and its derivatives (graphene oxide and reduced graphene oxide) have remarkable properties and they have been proposed for multiple applications such as adsorbents for dye separation [1], anti-corrosion coatings [2], drug delivery [3], desalination [4], and as a catalyst for hydrogen generation [5], among others. They exhibit a high specific area, abundant functional groups and sp^2^ hybridization, showing a high affinity to aromatic compounds. They are amenable to modification and functionalization by the appropriate introduction of the desired functionalities for different applications [6]. Moreover, since graphene nanomaterials exhibit excellent electrical and mechanical properties as well as high thermal conductivity, they have attracted widespread attention as electronic devices for solar cells, supercapacitors, and electrochemical sensors [7,8,9].

Specifically, graphene oxide (GO) contains oxygen functional groups such as epoxides, alcohols, and carboxylic acids, which are accessible for specific derivatization or functionalization through, for example, a one-pot synthesis [10,11] or an esterification reaction promoted with N,N0-dicyclohexylcarbodiimide (DCC) and N,N0- and dimethylaminopyridine (DMAP) [12,13,14] or using acetic anhydride and ethyl acetate as an eco-friendly solvent [15].

The combination with biomolecules, macrocycles or other nanomaterials seeks to improve the performance of graphene-based materials by extending the properties of both materials. Macrocyclic compounds such as cyclodextrins (CDs) have the ability to include lipophilic guest molecules into their cavities to form inclusion complexes. CDs are three-dimensional structures composed of D-glucopyranose units linked by α-(1 → 4) glucoside bonds. Among them, the most used is beta-cyclodextrin (βCD), which consists of seven glucopyranose units. Thus, conjugating CDs with graphene-based materials would provide a material with enhanced characteristics. For example, the functionalization of graphene oxide with CDs has been studied as a corrosion inhibitor for carbon steel in an acidic environment [16]. Given that the adsorption properties also improve, it can be used to adsorb heavy metals or organic contaminants in wastewater [17,18,19] or for the extraction of flavonoids [20].

Different methodologies to prepare a material combining graphene oxide or reduced graphene oxide (rGO) and cyclodextrins have recently been reported. Graphene oxide and βCD electrodes have been prepared by a simple electrooxidation process [21], using ammonia and hydrazine solutions [22,23,24], or by electropolymerization onto the rGO modified glassy carbon electrode (rGO/GCE) [25,26,27]. Graphene oxide has been epoxy-functionalized using epichlorohydrin, then βCD is incorporated under stirring at 60 °C for 3 h [17,28,29]. Rathour et al. prepared βCD-GO in basic media with NH_3_ solution under stirring for 5 h at 60 °C [30]. Yang et al. [31] also used a basic media following a procedure in three stages. The last stage involves heating at 60 °C during 3 h of reaction. Cao et al. used an amino-cyclodextrin derivative in DMF. The mixture was stirred at 60 °C for 12 h [32]. Amino-cyclodextrin derivatives with dicyclohexylcarbodiimide have been used to prepare a combined material with graphene oxide, stirring the mixture by 24 h at 80 °C [33]. Einafshar et al. used a carbodiimide compound (EDC) and 4-dimethyl-aminopyridine in a procedure that involved stirring for 4 days [18]. A hydrothermal method where the mixture was heated at 100 °C for 12 h was proposed by Huang et al. [34]. In previous works, βCD-GO nanosheets were formed after stirring overnight and then the reduction was promoted by adding NaBH_4_ and MnCl_2_ into the suspension that was subsequently heated at 90 °C for 1 h with constant stirring [35].

In this work, we intend to attach an amino-cyclodextrin derivative to graphene oxide using a simple methodology based on an esterification reaction previously used for immobilization on gold [36,37]. This procedure avoids the use of organic solvents such as hydrazine, or long reaction times and high temperatures. In this way, the oxygen-containing groups of graphene oxide could be activated using a carbodiimide (EDC) and hydroxysuccinimide (NHS), seeking the attachment of amino-cyclodextrin by the formation of amide bonds. The subsequent electroreduction will produce reduced graphene oxide (erGO) combined with cyclodextrin, aiming at applications in electrochemical sensors. As a proof-of-concept, the determination of the pesticide carbendazim was evaluated. Carbendazim is a widely used broad-spectrum benzimidazole fungicide applied in seed preplanting treatment and postharvest food storage. Additionally, it is applied repeatedly to control plant diseases including soilborne diseases over a growing season. The formation of the carbendazim-βCD inclusion complex was confirmed using a phase-solubility diagram and showed an increase of 57% of the solubility of carbendazim due to complexation [38]. Thus, this pesticide is a good candidate to check the functionalization and performance of the material containing both cyclodextrin and graphene oxide.

## 2. Experimental

### 2.1. Materials and Methods

Graphene oxide (GO, 4 mg/mL dispersion) and 6-monodeoxy-6-monoamino-β-cyclodextrin (namely, CD1, MW = 1134 g/mol free base) were purchased from Graphenea (Spain) and AraChem (The Netherlands), respectively. N-hydroxysuccinimide (NHS), N-(3-dimethylaminopropyl)-N¢-ethylcarbodimide (EDC), and carbendazim (methyl-1H-benzo-[d]-imidazol-2-yl-carbamate) were obtained from Sigma-Aldrich. Other chemicals were of analytical grade and used without further purification. All solutions were prepared with ultrapure water (18 MΩ cm) from a MilliQ system.

SEM images were obtained with a Field emission scanning electron microscope (FE-SEM), model INSPECT-F50, FEI (The Netherlands). SEM measurements were carried out using a glassy carbon disc (TED Pella Brand, Inc. (N16524) 12.7 mm diameter) modified with GO or CD1-GO. The ATR-FTIR characterization experiments were performed with an Interspec 200-X spectrometer with Insterspec for Windows 2000/3000/4000 version 3.40 Pro software. The Raman measurements were carried out with a Raman Advantage 532 DeltaNu spectrometer equipped with a 532 nm excitation laser line, with NuSpec DeltaNu 2009 software. The 10.0 mm diameter modified glassy carbon discs were placed directly under the laser beam of the RAMAN spectrometer. The spectra were scanned in the 1800–200 cm^−1^ region.

X-ray photoelectron spectroscopy (XPS) data were recorded under a vacuum better than 3 × 10^−9^ mbar using a PHOIBOS−150 (Specs) electron analyzer and Mg Kα radiation. The spectra were recorded using a constant pass energy of 20 eV and the binding energy scale was referred to the main C–C signal of the C 1s core level located at 284.6 eV.

Electrochemical measurements were carried out with a BAS CV-50 W potentiostat using a one-compartment electrochemical cell. A conventional three-electrode configuration was used in all electrochemical measurements. Glassy carbon electrodes (GCE, 3 mm diameter, model CHI104, CH Instruments), either bare or modified, were used as working electrodes. A platinum wire and Ag/AgCl, 3 M NaCl (BASi MF-2052) were used as auxiliary and reference electrodes, respectively. All potentials refer to the Ag/AgCl reference electrode. All electrochemical experiments were performed after bubbling the electrochemical cell with nitrogen for 10 min.

### 2.2. Preparation of Cyclodextrin Functionalized Graphene Oxide (CD1-GO)

**Scheme 1:**
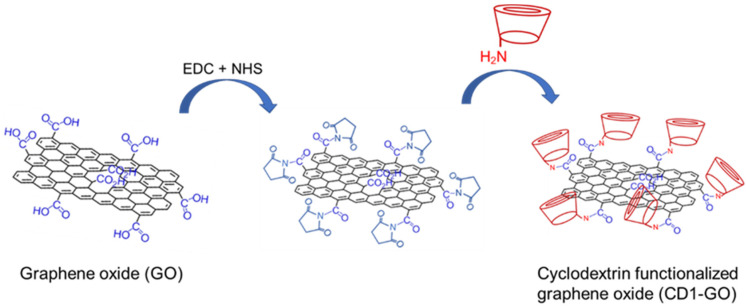
Schematic representation of the reactions in the preparation of CD1-GO.

The dispersion of GO (4 mg/mL) was used as received. A total of 1.5 mg of EDC and 2.4 mg of NHS were added to a 1 mL of GO aqueous dispersion and shaken for 20 min at room temperature (24–25 °C). Then, the product was centrifuged at 1500 rpm. The centrifuge product was transferred to 1 mL of CD1 solution (6 mM) under vigorous stirring for 20 min. The product was centrifugated and washed in ultrapure water several times to remove the unreacted CD1 and redispersed in 1 mL of water. The CD1-GO suspension was sonicated for 1 h before its use for electrode modification (see Scheme 1). 

### 2.3. Preparation of CD1-GO/GCE

The GCE electrode was polished with aluminum oxide particle sizes of 0.3 and 0.05 μm (Buehler) followed by profuse rinsing with ultrapure water and dried under a nitrogen stream. An aliquot of 5 μL of CD1-GO suspension was added on the electrode surface to form CD1-GO/GCE. The electrode was placed in an oven at 50 °C for 20 min. For comparative purposes, the same procedure was followed modifying GCE with GO to form GO/GCE.

### 2.4. Preparation of CD1-erGO/GCE

The prepared CD1-GO/GCE electrode was transferred to the electrochemical cell. The electroreduction process was carried out in a deaerated 0.1 M phosphate buffer solution (PBS, pH 7.0) by cyclic voltammetry (CV) according to previous procedures [39,40] from 0 to −1.5 V at 0.1 V/s. The optimal cycle number was studied previously by evaluating results from three to 50 cycles. Therefore, CD1-erGO/GCE was obtained. For comparative purposes, erGO/GCE (electrochemically reduced graphene oxide/glassy carbon electrode non-cyclodextrin functionalized) was also prepared following the same procedure. 

## 3. Results and Discussion

### 3.1. Morphological and Structural Characterization: SEM, ATR-FTIR, Raman, and XPS

**Figure 1:**
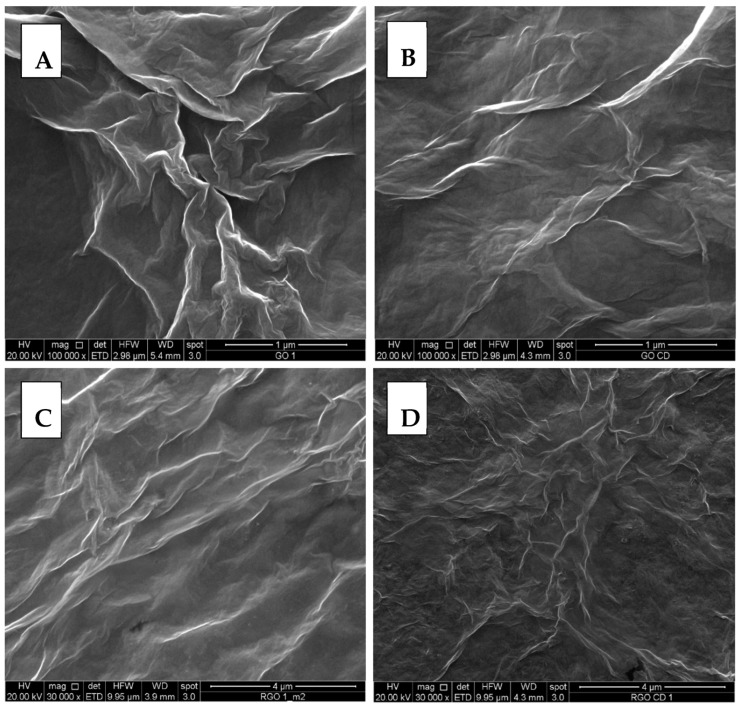
SEM images obtained for GO (**A**), CD1-GO (**B**), erGO (**C**), and CD1-erGO (**D**). Scale bar is 1 µm (**A**,**B**) and 4 µm (**C**,**D**). HV: 20.00 kV; Magnification: 100,000× (**A**,**B**) and 30,000× (**C**,**D**); HFW: 2.98 µm (**A**,**B**) and 9.95 µm (**C**,**D**).

The commercial GO and prepared erGO and CD1-erGO were characterized by SEM (Figure 1). The SEM image recorded from GO (Figure 1A) exhibited a typical morphology of GO with a wrinkled and folded texture showing irregular edges and crumpling, a reflection of its layered microstructure [31]. 

In the morphology images of CD1-erGO (Figure 1B), a smoother surface than that of GO was observed, showing a much more homogeneous distribution of layers after functionalization with CD1. There was a decrease in the crumpling and bending of sheets caused, most likely by the connection of CD1 and GO. This result differs from the one reported by Bhattacharjee et al. [41], probably due to differences in the preparation methodology used.

The images of erGO and CD1-erGO, taken with a smaller magnification, are also presented in Figure 1C,D. These images did not show significant differences before and after electroreduction, indicating that this process does not affect the network structure.

**Figure 2:**
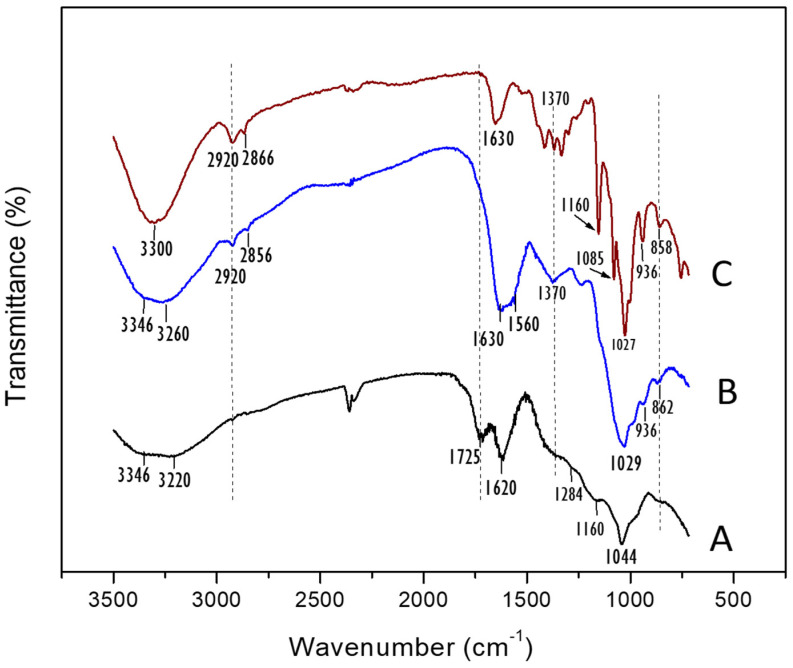
ATR-FTIR spectra of GO (**A**), CD1-GO (**B**), and CD1 (**C**).

The ATR-FTIR spectra recorded from commercial GO, CD1, and CD1-GO are shown in Figure 2. The GO spectrum showed a fairly broad peak in the region around ~3500 to 3000 cm^−1^ associated with stretching vibrations of the hydroxyl group. Furthermore, other characteristic bands were observed: C=O carboxyl stretching at 1725 cm^−1^, C–OH bending vibrating peak at 1620 cm^−1^, and C–O stretching vibration at 1044 cm^−1^ [42,43]. The signal corresponding to C–OH stretching vibration was associated with the shoulders observed at 1284 cm^−1^ and 1406 cm^−1^. The spectrum recorded from CD1 (Figure 2C) exhibited a characteristic peak at 3300 cm^−1^ due to the –OH group stretching in the primary and secondary hydroxyl groups. The N–H stretching vibration of the amine group of CD1, expected in the region 3300–3400 cm^−1^, was masked by the strong OH absorption. Two small bands associated with the antisymmetric and symmetric C–H stretching vibrations were observed at 2920 and 2866 cm^−1^. The bands observed at 1630 and 1370 cm^−1^ are usually assigned to H–O–H deformation modes of water molecules within the cavity. The band at 1630 cm^−1^ was slightly broad probably due to merging with the absorption band attributed to the N–H bending vibration of the –NH_2_ group of CD1 [44]. The bands observed at 1160, 1085, and 1027 cm^−1^ were associated with the C–C stretching vibration and the vibrations of C–O bonds in the ether and hydroxyl groups coupled with the asymmetric glycosidic vibration of C–O–C [45,46]. Two small additional bands appearing at 858 and 936 cm^−1^ were also observed, which were associated with the C–H deformation vibrations [45].

After the functionalization of GO with CD1 to form CD1-GO (Figure 2B), the band corresponding to the C=O carboxylic stretching vibration at 1725 cm^−1^ disappeared while a strong band, attributable to the C=O stretching vibration of the amide bond, was observed at 1630–1560 cm^−1^, which would be coupled with the bands corresponding to CD1 previously described.

Furthermore, the bands associated with CD1 were also observed in the spectrum of CD1-GO, although they were somewhat less intense and two of them appeared slightly shifted (in any case, the shift was small and of very little significance: 2866 → 2856 and 858 → 862 cm^−1^). The O–H band of CD1-GO observed in the range 3260–3346 cm^−1^ was stronger than that observed in GO. The bands at 1027 and 1044 cm^−1^ in the spectra of CD1 and GO, respectively, which belongs to the stretching vibration of C–O, appeared merged at 1029 cm^−1^ with a significant increase in intensity. 

These results confirm the effective functionalization to give CD1-GO. 

**Figure 3:**
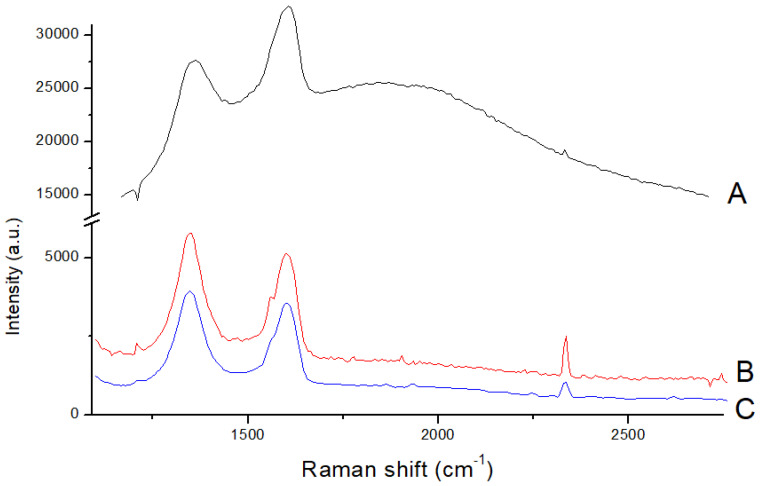
Raman spectra of GO (**A**), erGO (**B**), and CD1-erGO (**C**).

CD1-GO and GO were used to modify glassy carbon electrodes and an electrochemical process was applied to reduce GO and to restore the high electrical conductivity of graphene generating erGO/GCE and CD1-erGO/GCE (Figure 3). The Raman spectra were recorded to verify the formation of erGO. The Raman spectra of GO (Figure 3A) showed the characteristic D and G bands at 1362 and 1607 cm^−1^, related to the intensity of the sp^3^ defect and sp^2^ hybridization, respectively [47,48].

With the electroreduction process to give erGO (Figure 3B), these bands appeared at 1350 and 1600 cm^−1^ and the I_D_/I_G_ ratio was larger (1.13 ± 0.02) in comparison with that observed in the GO spectrum (0.82 ± 0.02). This increase in the I_D_/I_G_ ratio in the Raman spectrum of erGO has usually been interpreted as originating through an increase in the density of sp^3^ defects, and has been previously reported when electrochemical reduction has been used [49]. With the CD functionalization (CD1-erGO, Figure 3C), the I_D_/I_G_ ratio increased slightly from 1.13 to 1.36, with no changes in the band shifts. This change in the I_D_/I_G_ ratio suggests an increase in the sp^3^ structures due to the attachment of CD1. 

**Figure 4:**
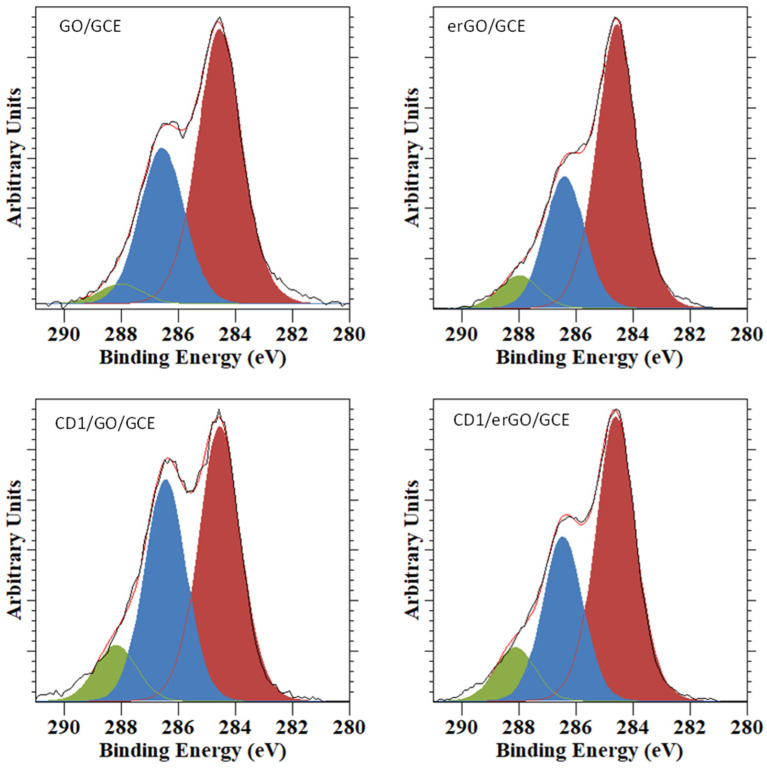
C 1s XPS spectra recorded from the different samples.

Figure 4 collects the C 1s XPS spectra recorded from the different samples. The spectrum of GO/GCE was similar to that reported previously for graphene oxide [1] and was fitted to three different contributions at 284.6 eV, 286.6 eV, and 288.1 eV. These contributions correspond to the C–C, C–O/C-OH, and C=O bonds, respectively [1]. These three contributions were also present in the spectra of the rest of the samples, but showed different relative spectral areas. In the case of the erGO/GCE sample, there was a significant decrease in the intensity of the contribution at 286.6 eV (C–O/C–OH) (29%) with respect to that shown in the spectrum of GO/GCE (34%), as can be expected for a material that has undergone reduction. The spectra of the CD-containing samples showed an increase in this same component (40% and 32%, respectively) with respect to their CD-free counterparts. This can be explained taking into account that the CDs contain numerous hydroxyl groups in their broader rim, and is therefore an indication that the CDs have been successfully deposited on the GO/GCE and erGo/GCE electrodes.

**Figure 5:**
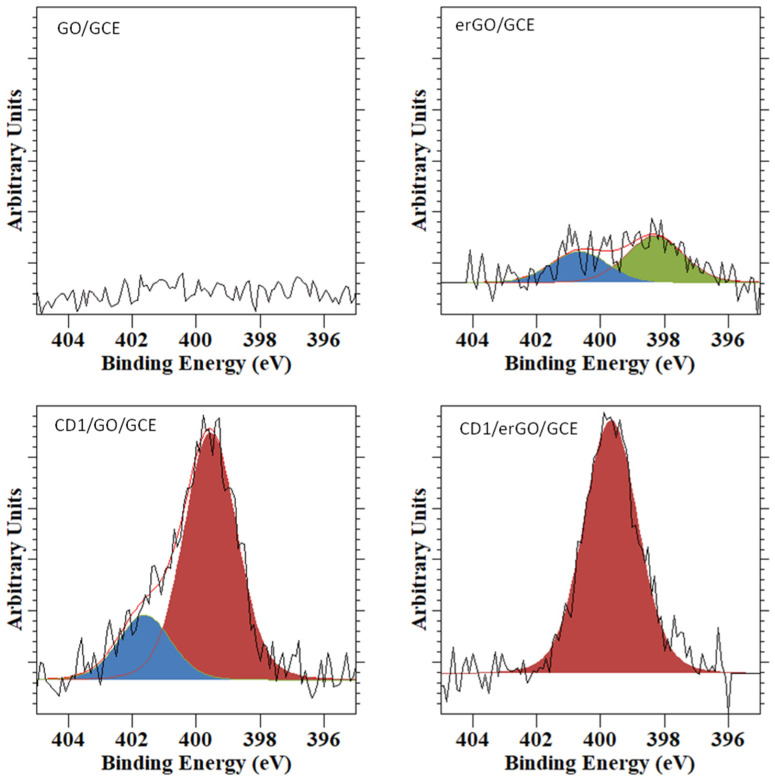
N 1s XPS spectra recorded from the different samples.

The attachment of the CDs to the electrode surfaces was further confirmed by the N 1s data (see Figure 5). The data recorded from GO/GCE indicate that this sample does not contain any surface nitrogen. The N 1s spectrum recorded from erGO/GCE showed a very small nitrogen contribution, which was fitted to two components located at 398.3 eV and 400.7 eV and can be associated to –NH_2_ amine bonds and –NHOH bonds, respectively [50]. We have reported in a previous publication that it is not unusual that glassy carbon contains nitrogen functionalities [51]. Therefore, the results suggest that these nitrogen species might arise from a fraction of the GCE, which could have not been completely covered by the erGO. The N 1s spectra recorded from the CD-containing electrodes were significantly more intense. The sample CD1/GO/GCE showed two different nitrogen species, a more intense one located at 399.6 eV, characteristic of amide groups, and a less intense one at 401.7 eV which corresponded to quaternary nitrogen [51]. The N 1s spectrum recorded from CD1/erGO/GCE contained only one amide contribution at 399.7 eV. The occurrence of amide bonds in these two last samples strongly suggests that the CDs had been covalently attached to the electrode surfaces.

In summary, both the C 1s and N 1s spectra were consistent with the successful attachment of the CDs units, via amide covalent bonds, to the GO and erGO/GCE electrodes.

### 3.2. Electrochemical Performance of CD1-erGO

**Figure 6:**
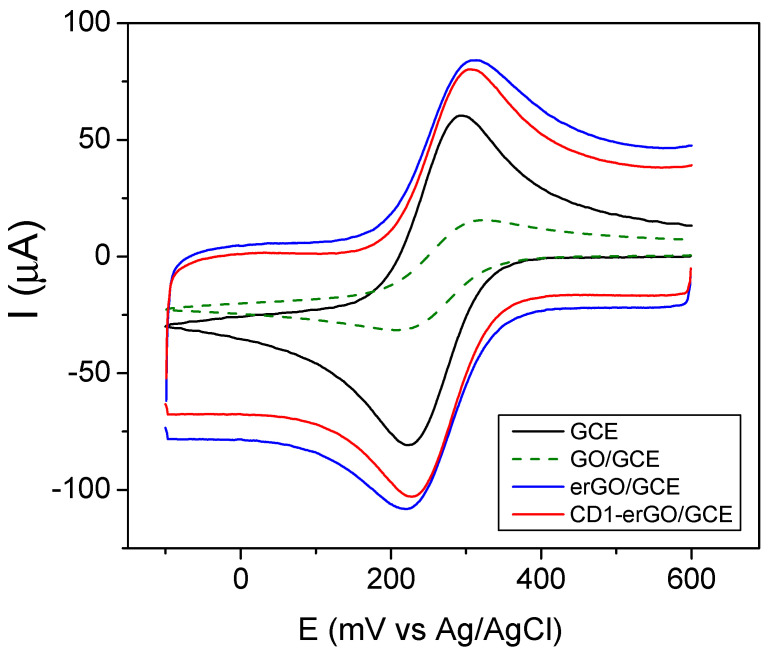
Cyclic voltammograms obtained in 5.0 mM K_3_[Fe(CN)_6_] in 0.5 M KCl on bare GCE (black line), GO/GCE (dashed green line), erGO/GCE (blue line), and CD1-erGO/GCE (red line). Scan rate: 0.1 V s^−1^.

The redox probe Fe(CN)_6_^3−/4−^ was used to evaluate the electrochemical behavior of both erGO/GCE and CD1-erGO/GCE (Figure 6). The corresponding comparison with bare GCE was also carried out. The typical cyclic voltammogram of ferricyanide at GCE was observed with a peak potential separation (ΔEp) of 78 ± 1 mV. GO/GCE showed the lowest current density due to its low electrical conductivity [52]. erGO/GCE showed a larger peak current than GO/GCE and bare GCE together with a slightly smaller ΔEp (69 ± 2 mV). The electrochemical reduction process removes the oxygen functional groups present in GO, restoring its conductivity. This characteristic behavior [53] showed that a larger electroactive surface area and a faster electron transfer were obtained with erGO/GCE. These changes confirm the effective electroreduction of GO on the GCE surface. 

Upon functionalization with CD1 to give CD1-erGO/GCE, there was a small decrease in the current density, indicating an increase in the electron transfer resistance, as previously verified by Ghanbari et al. using electrochemical impedance [27].

The electrochemical surface area of the modified electrodes can be estimated by using the Randles-Sevcik equation [54] (Equation (1)) utilizing cyclic voltammetry with 1.0 mM K_3_[Fe(CN)_6_] as a test solution and 0.1 M KCl as the supporting electrolyte, at different sweep rates. At T = 298 K and for a reversible process, the equation relating the current intensity with the area of the electrode surface is given by:

(1) Ip=2.69×105n32AD12C0v12

where *Ip* is the peak current; *n* is the number of electrons transferred; *A* is the electrochemical area; *C*_0_ is the concentration of K_3_[Fe(CN)_6_]; *D* is the diffusion coefficient (7.6 × 10^−6^ cm^2^ s^−1^); *ν* is the scan rate. The values are shown in Table 1.

A significant decrease of 60% of the electroactive area was observed when the glassy carbon electrode was modified with GO, showing an insulating behavior of GO. The sp^2^ hybridization of the graphene lattice with defects containing oxygen functionalities can block the electron pathways [55], making difficult the electrochemical reaction of the redox mediator. As it can be seen, the electrical conductivity was restored by the electrochemical reduction process. Thus, erGO/GCE showed an electroactive area of 0.093 cm^2^ (i.e., 3.5 times the electroactive area of GO/GCE). The electroactive areas with CD1 were lower than with the corresponding non-functionalized electrodes. This result was expected because the CD1 molecules block the redox reaction of ferricyanide since there is no formation of inclusion complexes of ferricyanide with cyclodextrins. Therefore, the decrease in the electroactive area of CD1-GO/GCE and CD1-erGO/GCE compared with GO/GCE and erGO/GCE, respectively, would indicate the presence of CD1 on the surfaces. 

Intra-day and inter-day reproducibilities were studied by cyclic voltammetry using ferricyanide anion as the redox probe. Five different CD1-GO/GCE electrodes were prepared separately and evaluated for five different measurements of 0.5 mM K_3_[Fe(CN)_6_] in 0.1 M phosphate buffer + KCl 0.1 M. Five different erGO/GCE electrodes were also prepared. The results showed that practically the same electrochemical response of ferricyanide was obtained for all electrodes with a relative standard deviation (RSD) lower than 8.5% for both CD1-erGO/GCE and erGO/GCE, showing an acceptable reproducibility of the electrodes. The electrochemical stability of the cyclodextrin-functionalized GO (CD1-GO) was studied by storing the suspension for one month and using it after that time to modify five glassy carbon electrodes to form CD1-GO/GCE electrodes, which were evaluated using ferricyanide anion in the same conditions and following the same reproducibility protocol. Furthermore, three CD1-GO/GCE modified electrodes were stored at room temperature for one week and then used to measure the ferricyanide solution. The results showed that the peak current values retained 95.2% of its initial value, showing a considerable stability of the modified electrodes.

**Scheme 2:**
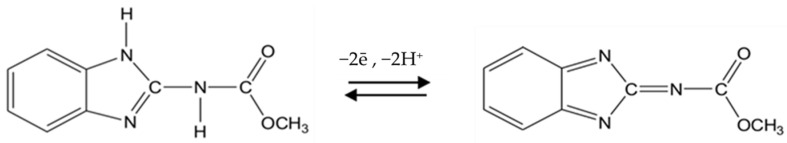
Proposed electrooxidation reaction of carbendazim.

**Figure 7:**
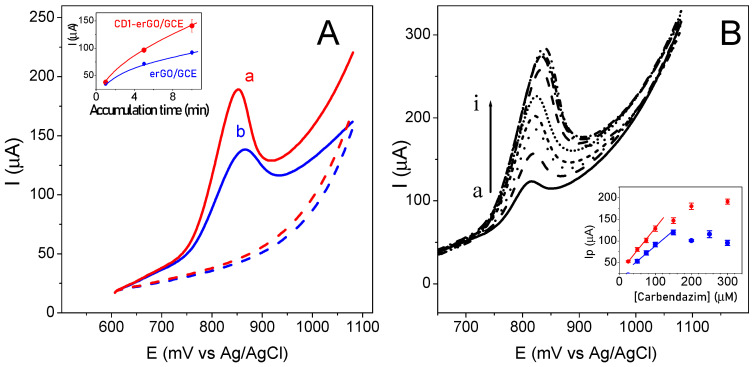
(**A**): Linear scan voltammograms obtained for 0.1 mM carbendazim in 0.1 M PBS (pH 7.0) at erGO/GCE (a) and CD1-erGO/GCE (b) using an accumulation time of 10 min. Baseline is shown as a dotted line in each case. Inset: Influence of the accumulation time of carbendazim at erGO/GCE (blue) and CD1-erGO/GCE (red) on the carbendazim oxidation peak current. (**B**) Linear scan voltammograms obtained for carbendazim at CD1-erGO/GCE using an accumulation time of 10 min. Carbendazim concentrations ranged from 25 to 400 μM (named from a to i). Inset: Effect of carbendazim concentration at erGO/GCE (blue) and CD1-erGO/GCE (red) on the carbendazim oxidation peak current using an accumulation time of 10 min. Supporting electrolyte 0.1 M phosphate buffer solution, pH 7.0. Scan rate: 0.050 V s^−1^.

In order to evaluate the electrochemical response of CD1-erGO/GCE, we studied the oxidation of the carbendazim pesticide as proof-of-concept using linear scan voltammograms. For comparison, the electrochemical response of carbendazim at erGO/GCE was measured. Figure 7A displays the linear scan voltammograms of carbendazim on the electrodes. Basically, only one electrochemical oxidation peak was observed at both electrodes at pH 7.0. The proposed electrochemical oxidation process of carbendazim at this pH is shown in Scheme 2 [56].

CD1-erGO/GCE showed an enhanced oxidation activity compared with that of erGO/GCE. The oxidation peak on erGO/GCE was observed at 867 ± 3 mV, changing slightly to a lower potential value on CD1-erGO/GCE (Ep = 852 ± 2 mV). Taking into account that carbendazim can form inclusion complexes with cyclodextrin [38], the increase in the current would arise from the combination of the inclusion complex carbendazim-cyclodextrin, together with the fact that CD1 causes a decrease in the structure crumpling. 

The effect of the adsorption time of carbendazim at both electrodes on the oxidation current is shown in the inset of Figure 7A. The oxidation signal increased as the accumulation time increased. Saturation was observed over 25 min. Considering that a 50% increase in the current was registered with a 10 min accumulation time, the electrochemical experiments were carried out with this accumulation time. 

The electrochemical response of carbendazim at CD1-erGO/GCE (Figure 7B) showed a linear dependence of the oxidation current peak for concentrations of the pesticide in the range from 25 to 125 μM (Figure 7B inset), becoming independent and reaching a plateau for concentrations above 200 μM. Interestingly, when erGO/GCE was used, the current intensity decreased at carbendazim concentrations upon 150 μM under the same experimental conditions. The linear behavior was observed only from 50 to 150 μM. 

The calibration curves for CD1-erGO/GCE and erGO/GCE were described by the following regression equations: *Ip* [μA] = 1.01 [μA/μM] × C [μM] + 2.747 × 10^−5^ [μA] (r = 0.9993); *Ip* [μA] = 0.63 [μA/μM] × C [μM] + 4.07 × 10^−5^ [μA] (r = 0.9967), respectively. The limit of detection (LOD) and the limit of quantification (LOQ) of CD1-erGO/GCE, obtained from five runs, were 0.50 and 1.67 μM, respectively. These values were lower than those obtained with erGO/GCE with a LOD = 4.32 μM and LOQ = 14.40 μM.

The aim of this work was to show not only that a cyclodextrin-functionalized electrode (CD1-erGO/GCE) presented a lower detection limit and a better sensitivity than a non-functionalized one (erGO/GCE), but to show that the simple methodology described here to prepare it is valid to obtain a material with better sensitivity and higher detection limits, or at least comparable to those prepared using different procedures. Other methodologies involve either the use of organic solvents such as hydrazine [23,24], or long reaction times (12 h) [35] or the electropolymerization of CD on graphene oxide that has been chemically reduced following procedures that need more preparation steps as well as reagents such as acids and/or hydrazine [25,26]. The present methodology is simple and gives place to electrodes, showing significantly better performance than other materials obtained by more complex procedures (Table 2).

The previous table indicates that the LOD values obtained in this work were within the range of those reported for the best electrodes. We have also shown that higher currents can be measured with the CD1 functionalized electrode at low concentrations of carbendazim compared with a non-functionalized electrode, obtaining, as Table 2 clearly shows, a much larger sensitivity (1.01 and 0.63 μA/μM for CD1-erGO/GCE and erGO/GCE, respectively) than the other materials. 

The present results also demonstrate that CD1 can be effectively attached to GO using the simple proposed method, and that after a subsequent electroreduction process, it remains attached without bringing about a noticeable alteration in the morphology of the electrode surface.

## 4. Conclusions

We have reported a method to attach amino-CD derivatives to graphene oxide and reduced graphene oxide, obtained after the electroreduction of the former to give CD1-erGO, which can be used as a modified glassy carbon electrode. The data reported in the present work indicate that the combination of both the amino-CD derivative and graphene oxide produced a network that was not affected by a subsequent electroreduction process. The inclusion abilities of the attached CD, together with the electrical properties of reduced graphene oxide, produced an improved response of the modified electrode to the target analyte carbendazim. Thus, graphene oxide can be used as a platform to attach cyclodextrins by a simple method in which their inclusion abilities can be exploited.

## Figures and Tables

**Table 1:** Electrochemical area (EA) of the surfaces obtained by cyclic voltammetry with 1.0 × 10^−3^ mol/L K_3_[Fe(CN)_6_].

Surface	EA/cm^2^
GCE	0.068
GO/GCE	0.027
CD1-GO/GCE	0.022
erGO/GCE	0.093
CD1-erGO/GCE	0.088

**Table 2:** Comparison of CD1-erGO/GCE with other reported modified electrodes containing both cyclodextrin and reduced graphene oxide for different analytes.

Electrode	Sensitivity (μA μM^−1^)	Linear Range (μM)	LOD (μM)	Ref.
β-cyclodextrin functionalized reduced graphene oxide	0.0278	10–100	2.3	23
Reduced graphene oxide-β-cyclodextrin-platinum nanocomposite	0.011	40–170	0.12	24
Electropolymerization of β -cyclodextrin and Reduced Graphene Oxide	0.149	5–165	8.92	25
Electropolymerized β-cyclodextrin on rGO/Screen printed electrode	0.046	200–2000	67	26
Cyclodextrin-RGO/GCE	0.38	7.2–72.8	0.36	35
CD1-erGO/GCE	1.01	25–125	0.5	This work

## Data Availability

Not applicable.

## References

[1] Yao H., Yu H., Zhang B.Y., Chen K., Yi Q., Xie H., Hu X., Tang T., Cheng Y., Tao X. (2022). Approximately 1 nm-sized artificial tunnels in wrinkled graphene-graphene oxide composite membranes for efficient dye/dye separation and dye desalination. Chem. Eng. J..

[2] Zhang R., Yu X., Yang Q., Cui G., Li Z. (2021). The role of graphene in anti-corrosion coatings: A review. Constr. Build. Mater..

[3] Homem N.C., Miranda C.S., Teixeira M.A., Domingues J.M., Seibert D., Antunes J.C., Amorim M.T.P., Felgueiras H.P. (2022). Graphene oxide-based platforms for wound dressings and drug delivery systems: A 10 year overview. J. Drug. Deliv. Sci. Technol..

[4] Skuse C., Tarpani R.R.Z., Gorgojo P., Gallego-Schmid A., Azapagic A. (2023). Comparative life cycle assessment of seawater desalination technologies enhanced by graphene membranes. Desalination.

[5] Al-Azmi A., Keshipour S. (2022). New bidental sulfur-doped graphene quantum dots modified with gold as a catalyst for hydrogen generation. J. Colloid. Interface Sci..

[6] Hu H., Wen W., Ou J.Z. (2022). Construction of adsorbents with graphene and its derivatives for wastewater treatment: A review. Environ. Sci. Nano.

[7] Díez-Pascual A.M., Rahdar A. (2022). Graphene-Based Polymer Composites for Flexible Electronic Applications. Micromachines.

[8] Zhu C., Yang G., Li H., Du D., Lin Y. (2015). Electrochemical Sensors and Biosensors Based on Nanomaterials and Nanostructures. Anal. Chem..

[9] Woo Y.S. (2019). Transparent Conductive Electrodes Based on Graphene-Related Materials. Micromachines.

[10] Keshipour S., Mohammad-Alizadeh S., Razeghi M.H. (2022). Copper phthalocyanine@graphene oxide as a cocatalyst of TiO_2_ in hydrogen generation. J. Phys. Chem. Solids.

[11] Keshipour S., Mohammad-Alizadeh S. (2021). Nickel phthalocyanine@graphene oxide/TiO_2_ as an efficient degradation catalyst of formic acid toward hydrogen production. Sci. Rep..

[12] Keshipour S., Khezerloo M. (2019). Nanocomposite of hydrophobic cellulose aerogel/graphene quantum dot/Pd: Synthesis, characterization, and catalytic application. RSC Adv..

[13] Ahmadi H., Keshipour S., Ahour F. (2020). New water-soluble colorimetric pH and metal ione sensor based on graphene quantum dot modified with alizarine red S. Sci. Rep..

[14] Esmaeili M., Ahour F., Keshipour S. (2021). Sensitive and selective determination of trace amounts of mercury ions using a dimercaprol functionalized graphene quantum dot modified glassy carbon electrode. Nanoscale.

[15] Acocella M.R., D’Urso L., Maggio M., Avolio R., Errico M.E., Guerra G. (2017). Green and Facile Esterification Procedure Leading to Crystalline-Functionalized Graphite Oxide. Langmuir.

[16] Haruna K., Saleh T.A., Obot I., Umoren S.A. (2019). Cyclodextrin-based functionalized graphene oxide as an effective corrosion inhibitor for carbon steel in acidic environment. Prog. Org. Coat..

[17] Li L., Chen B., Su Z., Wu J., Pan Z., Fu Z. (2020). Preparation of cyclodextrin-modified graphene oxide and its adsorption properties for Pb (II). IOP Conf. Ser. Earth Environ. Sci..

[18] Einafshar E., Khodadadipoor Z., Nejabat M., Ramezani M. (2021). Synthesis, Characterization and Application of α, β, and γ Cyclodextrin-Conjugated Graphene Oxide for Removing Cadmium Ions from Aqueous Media. J. Polym. Environ..

[19] Tian H., Zeng H., Zha F., Tian H., Chang Y. (2020). Synthesis of Graphene Oxide–Supported β-Cyclodextrin Adsorbent for Removal of p-Nitrophenol. Water Air Soil. Pollut..

[20] Hou X., Lu X., Niu P., Tang S., Wang L., Guo Y. (2018). β-Cyclodextrin-modified three-dimensional graphene oxide-wrapped melamine foam for the solid-phase extraction of flavonoids. J. Sep. Sci..

[21] Urcuk A., Karadurmus L., Bakirhan N.K., Ozkan S.A. (2021). Enhancement of graphene oxide through β-cyclodextrin composite to sensitive analysis of an antidepressant: Sulpiride. Open. Chem..

[22] Mirzaei B., Zarrabi A., Noorbakhsh A., Amini A., Makvandi P. (2021). A reduced graphene oxide-b-cyclodextrin nanocomposite-based electrode for electrochemical detection of curcumin. RSC Adv..

[23] Fu L., Lai G., Yu A. (2015). Preparation of β-cyclodextrin functionalized reduced graphene oxide: Application for electrochemical determination of paracetamol. RSC Adv..

[24] Singh M., Jaiswal N., Tiwari I., Foster C.W., Banks C.E. (2018). A reduced graphene oxide-cyclodextrin-platinum nanocomposite modified screen printed electrode for the detection of cysteine. J. Electroanal. Chem..

[25] Oliveira A.E.F., Bettio G.B., Pereira A.C. (2018). An Electrochemical Sensor Based on Electropolymerization of ß-Cyclodextrin and Reduced Graphene Oxide on a Glassy Carbon Electrode for Determination of Neonicotinoids. Electroanalysis.

[26] Qin Q., Bai X., Hua Z. (2016). Electropolymerization of a conductive β-cyclodextrin polymer on reduced graphene oxide modified screen-printed electrode for simultaneous determination of ascorbic acid, dopamine and uric acid. J. Electroanal. Chem..

[27] Ghanbari M.H., Shahdost-Fard F., Khoshroo A., Rahimi-Nasrabadi M., Ganjali M.R., Wysokowski M., Rębiś T., Żółtowska-Aksamitowska S., Jesionowski T., Rahimi P. (2019). A nanocomposite consisting of reduced graphene oxide and electropolymerized β-cyclodextrin for voltammetric sensing of levofloxacin. Microchim. Acta.

[28] Mansori G., Gholivand M.-B., Es’Haghi Z. (2020). Es’haghi. β-cyclodextrin coated graphene oxide nanoadsorbents on the hollow fiber-solid phase microextraction and electrochemical monitoring of acetaminophen. Desalin. Water Treat..

[29] Wang S., Li Y., Fan X., Zhang F., Zhang G. (2015). β-Cyclodextrin functionalized graphene oxide: An efficient and recyclable adsorbent for the removal of dye pollutants. Front. Chem. Sci. Eng..

[30] Rathour R.K.S., Bhattacharya J., Mukherjee A. (2019). β-Cyclodextrin conjugated graphene oxide: A regenerative adsorbent for cadmium and methylene blue. J. Mol. Liq..

[31] Yang Z., Liu X., Liu X., Wu J., Zhu X., Bai Z., Yu Z. (2021). Preparation of β-cyclodextrin/graphene oxide and its adsorption properties for methylene blue. Colloid. Surf. B-Biointerfaces.

[32] Cao X.T., Showkat A., Kang I., Gal Y.-S., Lim K.T. (2016). β-Cyclodextrin Multi-Conjugated Magnetic Graphene Oxide as a Nano-Adsorbent for Methylene Blue Removal. J. Nanosci. Nanotechnol..

[33] Wu H., Peng J., Wang S., Xie B., Lei L., Zhao D., Nie H. (2015). Fabrication of graphene oxide-b-cyclodextrin nanoparticle releasing doxorubicin and topotecan for combination chemotherapy. Mater. Technol..

[34] Huang Q., Li M., Wang L., Yuan H., Wang M., Wu Y., Li T. (2018). Synthesis of novel cyclodextrin-modified reduced graphene oxide composites by a simple hydrothermal method. RSC Adv..

[35] Liu Z., Ma X., Zhang H., Lu W., Ma H., Hou S. (2012). Simultaneous Determination of Nitrophenol Isomers Based on βCyclodextrin Functionalized Reduced Graphene Oxide. Electroanalysis.

[36] García M., Bollo S., Rivas G.A., Ferreyra N.F., Yáñez C. (2016). Bottom up approaches for amino β-CD adsorption on gold surfaces. A comparative study. Electrochim. Acta.

[37] Méndez-Torres A.M., Sandoval-Altamirano C., Sánchez-Arenillas M., Marco J.F., Yáñez C. (2018). Amino β-cyclodextrins immobilized on gold surfaces: Effect of substituents on host-guest interactions. Electrochim. Acta.

[38] Lezcano M., Al-Soufi W., Novo M., Rodríguez-Núñez E., Tato J.V. (2002). Complexation of Several Benzimidazole-Type Fungicides with α- and β-Cyclodextrins. J. Agric. Food Chem..

[39] Shao Y., Wang J., Engelhard M., Wang C., Lin Y. (2010). Facile and controllable electrochemical reduction of graphene oxide and its applications. J. Mater. Chem..

[40] Jiang Y., Lu Y., Li F., Wu T., Niu L., Chen W. (2012). Facile electrochemical codeposition of “clean” graphene–Pd nanocomposite as an anode catalyst for formic acid electrooxidation. Electrochem. Commun..

[41] Bhattacharjee T., Rahman S., Deka D., Purkait M.K., Chowdhury D., Majumdar G. (2022). Synthesis and characterization of exfoliated beta-cyclodextrin functionalized graphene oxide for adsorptive removal of atenolol. Mater. Chem. Phys..

[42] Dreyer D.R., Park S., Bielawski C.W., Ruoff R.S. (2010). The chemistry of graphene oxide. Chem. Soc. Rev..

[43] Stankovich S., Piner R.D., Nguyen S.T., Ruoff R.S. (2006). Synthesis and exfoliation of isocyanate-treated graphene oxide nanoplatelets. Carbon.

[44] Potter C.F., Russell N.R., McNamara M. (2006). Spectroscopic Characterisation of Metallo-Cyclodextrins for Potential Chiral Separation of Amino Acids and L/D-DOPA. J. Incl. Phenom. Macrocycl. Chem..

[45] Zhang Y., Xie Q., Xia Z., Gui G., Zhang P., Meng L., Pan L. (2022). Amino β-cyclodextrin-functionalized GS/MWCNTs for simultaneous electrochemical determination of p-aminophenol and acetaminophen. Int. J. Electrochem. Sci..

[46] Savic-Gajic I., Savic I.M., Nikolic V.D., Nikolic L.B., Popsavin M.M., Kapor A.J. (2016). Study of the solubility, photostability and structure of inclusion complexes of carvedilol with b-cyclodextrin and (2-hydroxypropyl)-b-cyclodextrin. J. Incl. Phenom. Macrocycl. Chem..

[47] Kim K., Coh S., Tan L.Z., Regan W., Yuk J.M., Chatterjee E., Crommie M.F., Cohen M.L., Louie S.G., Zettl A. (2012). Raman spectroscopy study of rotated double layer graphene: Misorientation-angle dependence of electronic structure. Phys. Rev. Lett..

[48] Cançado L.G., Jorio A., Ferreira E.H.M., Stavale F., Achete C.A., Capaz R.B., Moutinho M.V.O., Lombardo A., Kulmala T.S., Ferrari A.C. (2011). Quantifying defects in graphene via Raman spectroscopy at different excitation energies. Nano Lett..

[49] Lim C.S., Chua C.K., Pumera M. (2014). Detection of biomarkers with graphene nanoplatelets and nanoribbons. Analyst.

[50] Urzúa J., Carbajo J., Yáñez C., Marco J.F., Squella J.A. (2016). Electrochemistry and XPS of 2,7-dinitro-9-fluorenone immobilized on multi-walled carbon nanotubes. J. Solid. State Electrochem..

[51] Silva K., Marco J., Yañez C. (2019). Covalent Immobilization of Amino-β-Cyclodextrins on Glassy Carbon Electrode in Aqueous Media. J. Electrochem. Soc..

[52] Li B., Pan G., Avent N.D., Lowry R.B., Madgett T.E., Waines P.L. (2015). Graphene electrode modified with electrochemically reduced graphene oxide for label-free DNA detection. Biosens. Bioelectron..

[53] Castro K.L.S., Oliveira S.M., Curti R.V., Araújo J.R., Sassi L.M., Almeida C.M., Ferreira E.H.M., Archanjo B.S., Cabral M.F., Kuznetsov A. (2018). Electrochemical Response of Glassy Carbon Electrodes Modified using Graphene Sheets of Different Sizes. Int. J. Electrochem. Sci..

[54] Bard A.J., Faulkner L.R. (2001). Electrochemical Methods, Fundamentals and Applications.

[55] Zhao J., Pei S., Ren W., Gao L., Cheng H.-M. (2010). Efficient preparation of large-area graphene oxide sheets for transparent conductive films. ACS Nano.

[56] Ilager D., Malode S.J., Shetti N.P. (2022). Development of 2D graphene oxide sheets-based voltammetric sensor for electrochemical sensing of fungicide, carbendazim. Chemosphere.

